# Heterogeneity of Treatment Effects for Intensive Blood Pressure Therapy by Individual Components of FRS: An Unsupervised Data-Driven Subgroup Analysis in SPRINT and ACCORD

**DOI:** 10.3389/fcvm.2022.778756

**Published:** 2022-02-03

**Authors:** Yaqian Wu, Jianling Bai, Mingzhi Zhang, Fang Shao, Honggang Yi, Dongfang You, Yang Zhao

**Affiliations:** ^1^Department of Biostatistics, Nanjing Medical University, Nanjing, China; ^2^Key Laboratory of Medical Big Data Research and Application, Nanjing Medical University, Nanjing, China; ^3^Jiangsu Provincial Key Laboratory of Biomarkers of Cancer Prevention and Control, Nanjing Medical University, Nanjing, China; ^4^Collaborative Innovation Center for Cancer Personalized Medicine, Nanjing Medical University, Nanjing, China; ^5^Key Laboratory of Modern Toxicology, Nanjing Medical University, Nanjing, China

**Keywords:** framingham risk score, cardiovascular diseases, self-organizing map, heterogeneous treatment effects, SPRINT, ACCORD

## Abstract

**Background:**

Few studies have answered the guiding significance of individual components of the Framingham risk score (FRS) to the risk of cardiovascular disease (CVD) after antihypertensive treatment. This study on the systolic blood pressure intervention trial (SPRINT) and the Action to Control Cardiovascular Risk in Diabetes blood pressure trial (ACCORD-BP) aimed to reveal previously undetected association patterns between individual components of the FRS and heterogeneity of treatment effects (HTEs) of intensive blood pressure control.

**Methods:**

A self-organizing map (SOM) methodology was applied to identify CVD-risk-specific subgroups in the SPRINT (*n* = 8,773), and the trained SOM was utilized directly in 4,495 patients from the ACCORD. The primary endpoints were myocardial infarction (MI), non-myocardial infarction acute coronary syndrome (non-MI ACS), stroke, heart failure (HF), death from CVD causes, and a primary composite cardiovascular outcome. Cox proportional hazards models were then used to explore the potential heterogeneous response to intensive SBP control.

**Results:**

We identified four SOM-based subgroups with distinct individual components of FRS profiles and the CVD risk. For individuals with type 2 diabetes mellitus (T2DM) in the ACCORD or without diabetes in the SPRINT, subgroup I characterized by male with the lowest concentrations for total cholesterol (TC) and high-density lipoprotein (HDL) cholesterol measures, experienced the highest risk for major CVD. Conversely, subgroup III characterized by a female with the highest values for these measures represented as the lowest CVD risk. Furthermore, subgroup II, with the highest systolic blood pressure (SBP) and no antihypertensive agent use at baseline, had a significantly greater frequency of non-MI ACS under intensive BP control, the number needed to harm (NNH) was 84.24 to cause 1 non-MI ACS [absolute risk reduction (ARR) = −1.19%; 95% CI: −2.08, −0.29%] in the SPRINT [hazard ratio (HR) = 3.62; 95% CI: 1.33, 9.81; *P* = 0.012], and the NNH of was 43.19 to cause 1 non-MI ACS (ARR = −2.32%; 95% CI: −4.63, 0.00%) in the ACCORD (HR = 1.81; 95% CI: 1.01–3.25; *P* = 0.046). Finally, subgroup IV characterized by mostly younger patients with antihypertensive medication use and smoking history represented the lowest risk for stroke, HF, and relatively low risk for death from CVD causes and primary composite CVD outcome in SPRINT, however, except stroke, a low risk for others were not observed in ACCORD.

**Conclusion:**

Similar findings in patients with hypertensive with T2DM or without diabetes by multivariate subgrouping suggested that the individual components of the FRS could enrich or improve CVD risk assessment. Further research was required to clarify the potential mechanism.

## Introduction

High blood pressure (BP) is a leading global preventable contributor to premature mortality worldwide, affecting more than one billion adults, primarily because of its association with the increased risk of cardiovascular disease (CVD). BP-lowering treatment can reduce the CVD risk, although the optimal treatment target for systolic blood pressure (SBP) remains widely debated (1). For example, in the SBP intervention trial (SPRINT), over a median follow-up of 3.26 years, patients randomly assigned to the intensive-treatment group (target SBP < 120 mmHg or standard-treatment goal < 140 mm Hg) obtained both clinically and statistically significant reductions in primary CVD [hazard ratio (HR) = 0.75; 95% CI: 0.64, 0.89] (2). Besides, an impressive reduction in CVD for the Empagliflozin Cardiovascular Outcome Trial also underscored the benefit of intensive BP control (target SBP < 130 mm Hg) in type 2 diabetes mellitus (T2DM) (3). However, the Action to Control Cardiovascular Risk in Diabetes blood pressure trial (ACCORD-BP), performed 9 years before the SPRINT and testing the same target SBP in patients with T2DM, failed to identify a significant protective effect on the CVD risk for intensive BP control (4). A *post-hoc* analysis by Basu et al. concluded that the differences in outcomes between the trials aforementioned of intensive BP treatment could be explained by the heterogeneity of the population (5).

Heterogeneous treatment effects (HTEs) denote whether the effects of intensive BP control are nonrandomly varied among patients with different clinical or genetic characteristics. Identifying such HTEs is clinically essential for the application of randomized trial data to patient care. In the SPRINT, several studies demonstrated HTEs, although the original trial did not report any subgroup heterogeneity. For example, Scarpa et al. revealed clinically significant heterogeneity associated with intensive BP control that current smokers with baseline SBP > 144 mm Hg had a significantly higher rate of the primary composite outcome under intensive BP treatment (6). Rostomian et al. found HTEs in the SPRINT that younger patients without chronic kidney disease (CKD) or CVD, or older patients with CKD or CVD could benefit from intensive BP control (7). In addition, Krishna et al. noted that intensive intervention was associated with a lower risk of CVD in patients with increasing age and higher baseline diastolic BP (DBP) and the opposite in patients with higher baseline SBP (8).

The Framingham Risk Score (FRS), developed from the Framingham Heart Study, has been widely used for predicting CVD risk over the past 10 years. The algorithm considers sex, age, total cholesterol (TC), high-density lipoprotein (HDL) cholesterol (HDL-C), SBP, antihypertensive agent use, smoking status, and diabetes status (9, 10). However, evidence that the effect of individual components of the FRS on the prevalence of incident cardiovascular events was different is substantial. For instance, Kozakova et al. have reported that prevalent cardiovascular events were independently associated with individual components of the FRS and, except for HDL-C, were all positively correlated with the CVD risk (11). Lee et al. revealed that BP and HDL-C had the greatest influence on the calculation of FRS, and the former was positively correlated with a 10-year CVD risk, while the latter was negatively related to the risk (12).

In this context, we do not question the role of FRS as the primary marker of increased CVD risk, but we aimed for a more comprehensive description of its components. Traditionally, subgroup analysis is applied to identify potential HTEs (13). However, such conventional one-variable-at-a-time analysis needs to be fully predefined and often fails to detect clinically meaningful HTE. Instead, a self-organizing map (SOM), an unsupervised data-driven subgrouping method, was applied to examine individual components of the FRS in BP treatment harms or benefits, using data from the SPRINT and ACCORD.

## Methods

### Study Population

The primary study sample included nondiabetic hypertensive patients with a high risk for CVD from the SPRINT, a large randomized, multicenter, and controlled study, conducted at 102 clinical sites in the United States between November 2010 and August 2015. A total of 9,361 participants were enrolled, with 4,678 randomized to the intensive-treatment group (target SBP < 120 mm Hg) and 4,683 randomized to the standard-treatment group (target < 140 mm Hg). Results from the SPRINT demonstrated that intensive control of hypertension would effectively reduce the risk of major cardiovascular events, including myocardial infarction (MI), nonmyocardial infarction acute coronary syndrome (non-MI ACS), stroke, heart failure (HF), or death from cardiovascular causes. Further details of SPRINT inclusion and exclusion criteria were available at the site: https://biolincc.nhlbi.nih.gov/studies/sprint/ (2).

Details on the design and results of the ACCORD study were available at https://biolincc.nhlbi.nih.gov/studies/accord/. Briefly, the ACCORD enrolled 4,733 participants with T2DM, who were randomly assigned to the same SBP-lowering target as in the SPRINT (14). Unlike the SPRINT, intensive antihypertensive therapy in the ACCORD did not significantly reduce the major cardiovascular outcomes.

### Endpoints

The primary CVD endpoint in the SPRINT defined for the current analysis included: nonfatal MI, non-MI ACS, nonfatal stroke, acute decompensated HF, death from CVD causes, and a primary composite cardiovascular outcome consisting of the aforementioned cardiovascular events.

The primary CVD in the ACCORD was similar to that of the SPRINT; however, it did not include acute decompensated HF or acute coronary syndrome. To match the SPRINT as closely as possible, we applied congestive HF or unstable angina outcome in the ACCORD as decompensated HF or acute coronary syndrome not resulting in myocardial infarction defined in SPRINT, and modified the definition of primary composite CVD outcome in the ACCORD (2, 15–18).

In this study, all the endpoints were analyzed using the identical analysis procedure individually.

### Individual Components of Framingham Risk Score

The FRS used in the SPRINT was derived initially from the Cox proportional hazards models in a 2008 paper by D'Agostino et al. using the seven variables as follows: sex, age, TC, HDL-C, SBP, antihypertensive medication use, and smoking status (10). Considering that participants with diabetes mellitus were excluded in the SPRINT, and the ACCORD only enrolled patients with type 2 diabetes, the variable diabetic state was not included. The identical components of FRS were selected in the ACCORD for validation. It was noteworthy that this research focused on the association between the individual components of the FRS and CVD, and intended to detect potential heterogeneity.

### Multivariate Subgrouping–Self-Organizing Map Analysis

**Figure 1:**
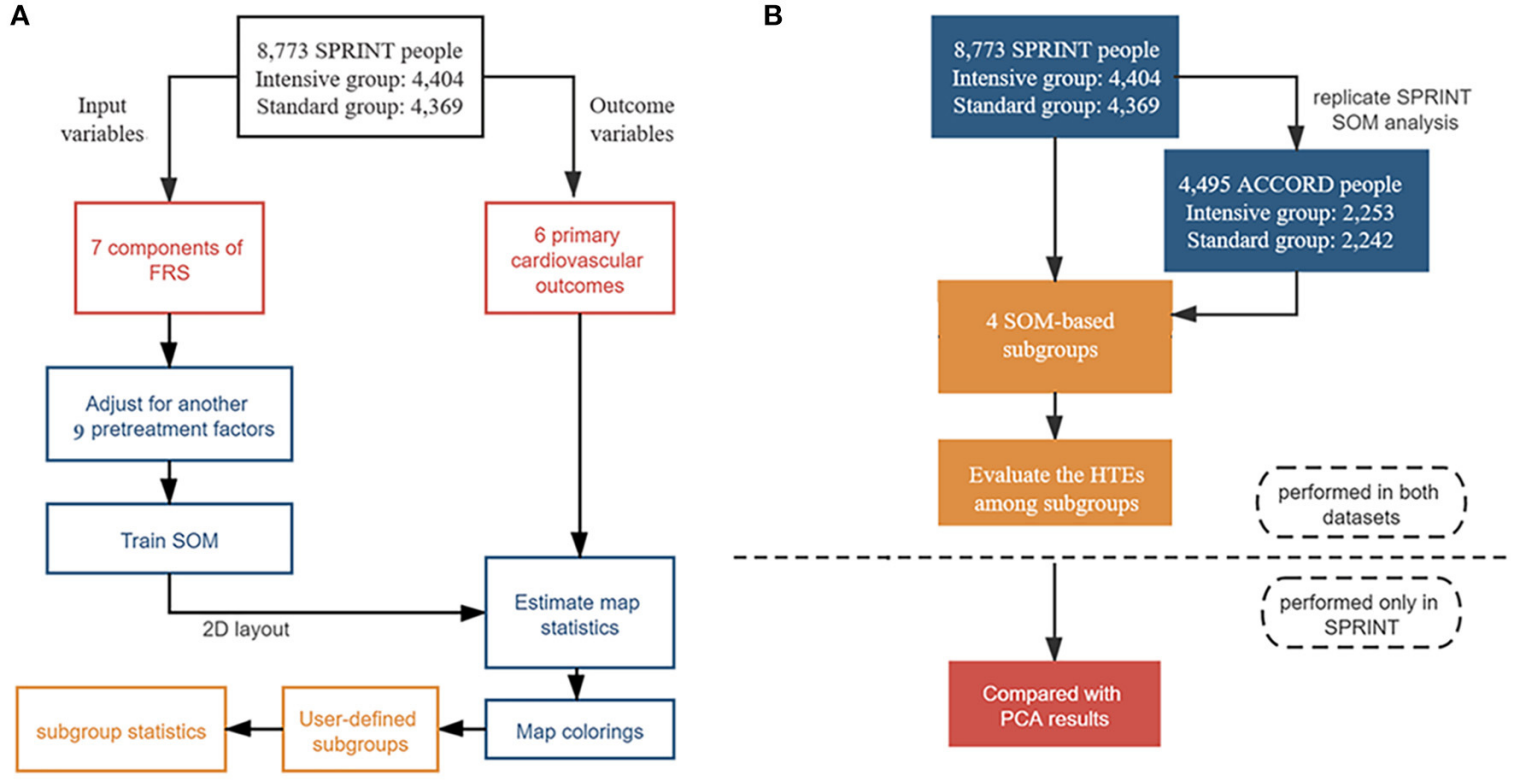
Flowchart of statistical analysis steps. **(A)** Steps of self-organizing map (SOM) analysis in the Systolic Blood Pressure Intervention Trial (SPRINT) data, including data preprocessing, SOM creating, map statistics estimating, maps coloring, interactive subgroup selection, and interpretation of the results process. **(B)** Steps of the overall analysis: SOM was trained only in the SPRINT and exploratory study of heterogeneity was conducted both in the SPRINT and ACCORD; further sensitivity analysis was also only performed in the SPRINT.

The R software package “Numero” was implemented to build SOMs. Conceptually, the SOM, an unsupervised pattern recognition method, was a projection of multidimensional data onto a two-dimensional map. Details of the SOM analysis are described elsewhere (19). As shown in Figure 1A, the SOM algorithm here was initialized based on a total of seven components of FRS only (collectively referred to as input or training variables). And, the distance on the map between two individuals corresponded to their similarity concerning the profiles for the components of the FRS that means those who shared similar profiles were located as close to each other as possible, whereas those who had different profiles were placed far apart on the map. For example, in this study each participant was assigned a location on the map based on seven components of FRS: people within the same map area shared a similar overall profile, while people far apart have different profiles (20). After positions of the individuals were computed, the map was colored according to training variables within different regions. Next, 20,000 random colorings were computed by permutation test to ensure the results were statistically reliable, where the null distributions were also the basis of the color scale in each map so that the categorical and continuous variables could be compared visually while maintaining the statistical interpretation. Finally, we could split the map into different subgroups from a multivariable perspective by colors of the regions, and then conventional statistics were used to evaluate the associations with primary endpoints.

It was noted that the SOM was based on a split-by-variable study design. In other words, the dataset was divided into two parts: the input set and the outcome set. We chose only the input variables (seven individual components of FRS) to construct the SOM. Since the outcome variables (six primary CVD outcomes) played no role in the training of the SOM, the statistical significance of the incidences of CVD events could be estimated without overestimating model accuracy.

### Statistical Analysis

Student's *t*-test or Pearson's chi-squared test was used to compare baseline participant characteristics between the intensive and standard BP control groups for SPRINT or ACCORD participants separately.

Self-organizing map analysis was first performed in the SPRINT to visually identify CVD risk-specific subgroups based on the combinations of individual components of the FRS, with adjustment for another nine pretreatment factors specified by cardiologists, including aspirin use, estimated glomerular filtration rate, glucose, triglycerides, urine albumin/creatinine ratio, body mass index (BMI), statin use, African American race, and subgroup with history of clinical/subclinical CVD. The trained SOM and the predefined subgroups were then directly applied in the ACCORD with an identical set of training variables to verify results from the SPRINT (21). Covariate balance in identified subgroups from these two cohorts between different BP treatment groups was evaluated using absolute mean standardized difference, with <0.1 considered as acceptable balance.

Cox proportional hazards regression analyses were used to estimate hazard ratios (HRs) and 95% CIs between intensive BP control and each cardiovascular outcome, with adjustment for sex, age, race, smoking status, body mass index (BMI), and use of antihypertensive agents. Cochrane's Q tests were then implemented to evaluate HTEs among SOM-based subgroups. For the heterogeneous cardiovascular endpoint, Kaplan–Meier curves were plotted to describe the cumulative hazard of the event over follow-up in each subset.

For external validation, the results of subgroup division from the SPRINT were directly used in the ACCORD since fully independent SOM training and subgrouping were performed solely in the SPRINT. The characteristics of the resulting SOM-based subgroups, and also the analysis of HTEs in the ACCORD, reached the same conclusion as in the SPRINT.

In addition, we performed two sensitivity analyses to explain the rationality of the visually SOM results: (i) two additional Cox proportional hazards regression models were used to assess the reliability of association results: Model 1 did not include any confounding factors; Model 2 was adjusted for TC, HDL-cholesterol and triglycerides; (ii) principal component analysis (PCA) was conducted, and the top three principal component scores and loadings were visualized to observe whether the same characteristics as identified in SOM-based subgroups could be captured.

Statistical analysis steps were shown in Figure 1. All the statistical analyses were performed using R 3.6.3. All tests were 2-sided, and the statistical significance threshold was 5%.

## Results

### Participant Characteristics

This analysis included 8,773 SPRINT participants (93.7% of the randomized participant sample; 4,404 or 4,369 individuals from the intensive or standard treatment arm) without nonmissing data on predictor variables and covariates. Descriptive statistics for baseline characteristics of the SPRINT participants included in this analysis were presented in Table 1.

**Table 1:** Baseline characteristics of the SPRINT and ACCORD participants included in the analysis.

**Characteristic**	**SPRINT** **(*****n*** **=** **8,773)**			**ACCORD-BP** **(*****n*** **=** **4,495)**		
	**Intensive group** **(*n* = 4,404)**	**Standard group** **(*n* = 4,369)**	***P* value**	**Intensive group** ***n* = 2,253)**	**Standard group** **(*n* = 2,242)**	***P* value**
Age, mean ± SD, y	67.9 ± 9.4	67.9 ± 9.5	0.953	62.7 ± 6.6	62.8 ± 6.8	0.803
Female sex, n (%)	1,577 (35.8)	1,524 (34.9)	0.376	1,068 (47.4)	1,060 (47.3)	0.957
SBP, mean ± SD, mm Hg	139.7± 15.8	139.7 ± 15.5	0.872	139.1 ± 16.1	139.4 ± 15.5	0.536
Antihypertensive agents, N (%)	4,000 (90.8)	3,958 (90.6)	0.734	1,954 (86.7)	1,914 (85.4)	0.204
Total cholesterol, mean ± SD, mg/dl	190.4 ± 41.7	189.6 ± 40.6	0.363	194.0 ± 45.1	191.4 ± 43.9	0.054
HDL cholesterol, mean ± SD, mg/dl	52.9 ± 14.4	52.7 ± 14.6	0.642	46.0 ± 13.2	46.3 ± 14.0	0.532
Smoking status, n (%)			0.503			0.959
Never	1,917 (43.5)	1,933 (44.2)	0.503	1,003 (44.5)	1,007 (44.9)	0.959
Former	1,875 (42.6)	1,865 (42.7)		949 (42.1)	940 (41.9)	
Current	612 (13.9)	571 (13.1)		301 (13.4)	295 (13.2)	
Aspirin use, n (%)	2,269 (51.5)	2,206 (50.5)	0.346	1,210 (53.7)	1,141 (50.9)	0.063
Estimated glomerular filtration rate, mean ± SD, mL/min/1.73 m^2^	71.6 ± 20.7	71.7 ± 20.7	0.896	91.6± 30.5	91.5 ± 27.3	0.912
Glucose, mean ± SD, mg/dL	98.9 ± 13.8	98.9 ± 13.4	0.992	176.1 ± 57.9	173.5 ± 57.8	0.120
Triglycerides, mean ± SD, mg/dL	125.5± 87.0	126.7 ± 81.0	0.507	195.4 ± 179.5	192.2 ± 173.3	0.551
Urine albumin in mg/(creatinine in g × 0.01), mean ± SD	43.7 ± 178.1	41.3 ± 153.9	0.502	85.7 ± 276.8	101.2 ± 370.1	0.111
Body mass index, mean ± SD, kg/m^2^	29.9 ± 5.8	29.8 ± 5.7	0.508	32.2 ± 5.6	32.1 ± 5.3	0.564
Statin use, n (%)	1,883 (42.8)	1,970 (45.1)	0.029^*^	1,448 (64.3)	1,486 (66.3)	0.166
African American race, n (%)	1,378 (31.3)	1,422 (32.5)	0.215	522 (23.2)	549 (24.5)	0.316
Subgroup with a history of clinical/subclinical CVD, n (%)	896 (20.3)	892 (20.4)	0.955	768 (34.1)	755 (33.7)	0.794

*Values were described as mean ± SD or number (%). p-values were based on ANOVA or Pearson's chi-squared tests*.

* *p-value < 0.05*.

*SPRINT, systolic blood pressure intervention trial; ACCORD, the action to control cardiovascular risk in diabetes; SBP, systolic blood pressure; HDL, high-density lipoprotein*.

### SOM-Based Subgrouping Analysis

**Figure 2:**
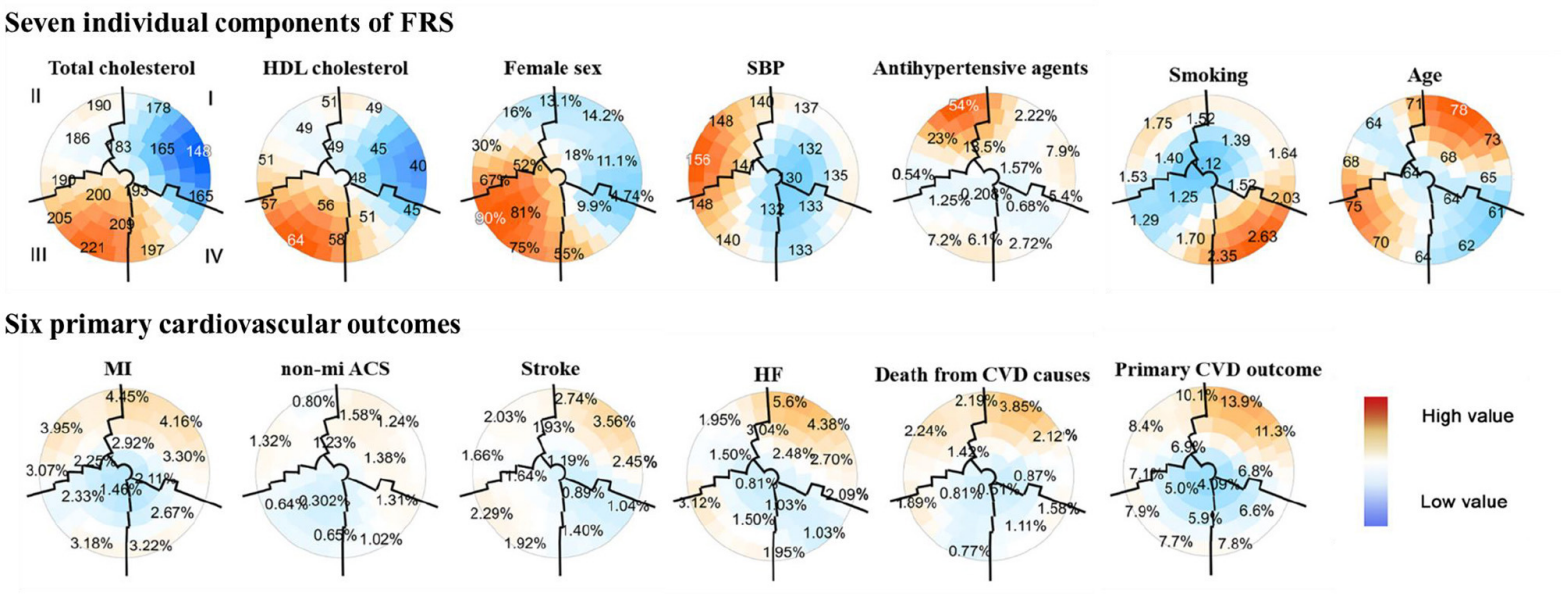
Subgrouping results from seven individual components of the FRS and six primary cardiovascular outcomes using the SOM in the SPRINT data (*n* = 8,773). Each component plane aforementioned showed coloring on the same SOM. The upper part of the two-dimensional SOMs were seven components of the FRS, including TC, HDL cholesterol, female, SBP, antihypertensive agents use, smoking status, and age. Six cardiovascular outcomes included MI, non-MI ACS, stroke, HF, death from CVD causes, and a composite cardiovascular outcome were in the lower part of the figure. The position for each participant on the map was unique and only dependent on the seven individual components of the FRS, so each participant was always located in the same place on each map. The color scale indicates the deviation from the population mean concerning the random fluctuations that could be expected by chance; dark red indicated the highest and dark blue for the lowest values. The numbers on the map represented the local mean value or prevalence (binary variables) for that region. Close districts were selected based on visual identification to provide relevant subgroups of individuals whose boundaries were marked by black lines. A total of four population subgroups were determined: subgroup I was characterized by men with the lowest TC and HDL-C, while subgroup III was women with the highest mean TC and HDL-C; subgroup II had the highest mean SBP with no antihypertensive medication use at the baseline visit, and subgroup IV characterized by mostly younger patients with antihypertensive medication use and smoking history. Patients with similar components of FRS were located close to each other throughout the map. SPRINT, systolic blood pressure intervention trial; FRS, framingham risk score; SOM, self-organizing maps; HDL, high-density lipoprotein cholesterol; SBP, sys tolic blood pressure; MI, myocardial infarction; non-MI ACS, nonmyocardial infarction acute coronary syndrome; HF, heart failure; CVD, cardiovascular disease.

Results from the subgrouping analysis using the SOM methodology in the SPRINT participants depicted in Figure 2 showed strong regional patterns, particularly for sex, TC, and HDL-C. For each SOM, patients identified as globally similar in terms of the seven components of FRS were located at fixed positions on the maps. This means that the participants were represented on the map according to the similarity of the seven components of the FRS, and a short distance indicated a remarkable resemblance, conversely, individuals with fewer commonalities are further apart. The districts were colored from blue to red, representing average district value levels from the lowest to highest (22, 23). For illustrative purposes, a selection of average district values was shown for representative districts in each SOM.

A total of four subgroups were visually identified for the SPRINT, labeled from I to IV with the boundaries overlaid using black lines, according to the following possible combinations between the seven FRS metrics: subgroup I (*n* = 2,874) in the upper right part of the maps, included a remarkably proportion of patients of CVD characterized by a profile of male with the lowest mean TC and HDL-C concentrations; subgroup III (*n* = 2,216) in the opposite corner was summarized by a profile of female with the highest levels of TC and HDL-C, conversely showed a low proportion of CVD; patients with the highest SBP and no antihypertensive agents use at baseline visit were located in the upper left area (subgroup II; *n* = 2,048); and subgroup IV comprised mostly younger patients with antihypertensive medication use and smoking history (*n* = 1,635; lower right place). Besides, the highest and lowest CVD prevalence was observed for subgroups I and III, respectively (Figure 2).

Detailed characteristics of the four SOM-based subgroups with a mean (SD) or number (percentage) were illustrated in Supplementary Table 1, along with Supplementary Figure 1 which indicated an overall good covariate balance between different BP treatment arms in those subgroups, since all absolute mean standardized difference of covariates <0.1.

The incidence of primary endpoints was investigated within each subgroup from the SPRINT (Supplementary Table 3). In accordance with SOM results, subgroup I had the significantly highest CVD prevalence (overall vs. subgroup I, 3.0 vs. 3.5% for MI; 1.0 vs. 1.3% for non-MI ACS; 1.9 vs. 2.4% for stroke; 2.3 vs. 3.7% for HF; 1.6 vs. 2.1% for death from causes of CVD; and 8.0 vs. 10.1% for primary CVD outcome). Contrary to subgroup I, subgroup III had the lowest incidence for the most cardiovascular events (overall vs. subgroup I, 3.0 vs. 2.4% for MI; 1.0 vs. 0.5% for non-MI ACS; 1.6 vs. 0.9% for death from CVD causes; and 8.0 vs. 6.4% for primary CVD outcome). Subgroup IV represented the lowest risk for stroke (overall vs. subgroup IV, 1.9 vs. 1.1%) and HF (2.3 vs. 1.4%), and relatively low risk for death from CVD causes (1.6 vs. 1.2%) and primary CVD outcome (8.0 vs. 7.0%) in the SPRINT, however, except stroke (1.9 vs. 1.3%), a low risk for others were not observed in the ACCORD (Supplementary Table 4).

### Exploratory Analysis of HTE

**Figure 3:**
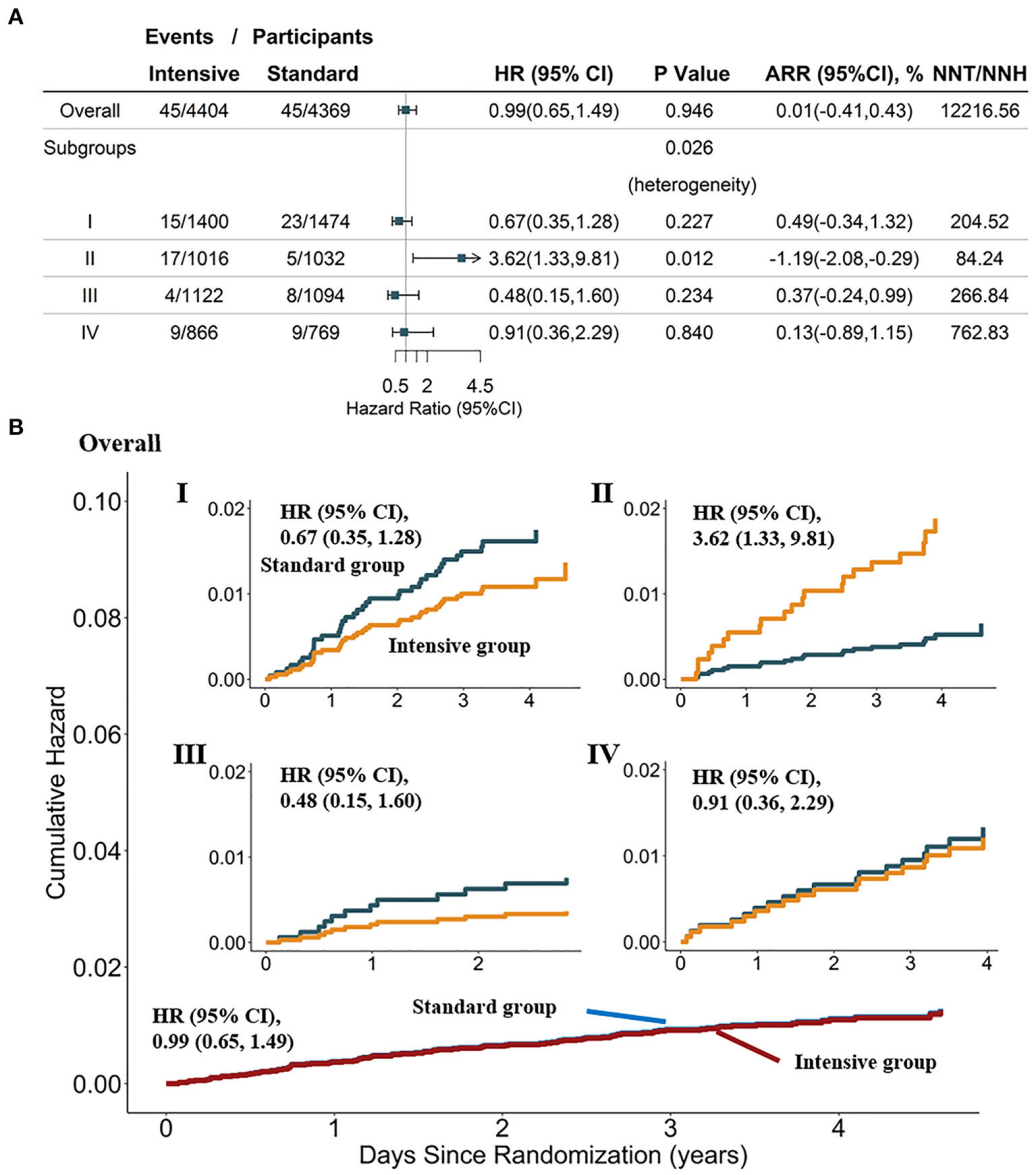
Heterogeneity analysis and cumulative hazard curves for non-MI ACS in the overall population and four SOM-based subgroups in the SPRINT dataset (*n* = 8,773). **(A)** Results from Cox analysis and heterogeneity analysis of four SOM-based subgroups, with adjustment for age, female sex, smoking status, BMI, statin use, and race; absolute risk reduction (ARR) = CVD incidence in the standard-treatment group—CVD incidence in the intensive-treatment group. **(B)** Cumulative hazard curves across intensive and standard treatment groups, adjustment for age, female, smoking status, BMI, statin use, race. SPRINT, systolic blood pressure intervention trial; SOM, self-organizing maps; non-MI ACS, non-myocardial infarction acute coronary syndrome.

Results from Cox proportional hazards regression in the SPRINT for the SOM-based subgroups (Supplementary Figure 2A) showed that consistent with the conclusions from SPRINT, individuals could benefit from intensive BP treatment in most cases (HR < 1, and 95% CI not crossing 1). In addition, along with the cumulative hazard curves, we revealed the HTEs of the intensive SBP control on non-MI ACS (Cochrane's Q tests *P* = 0.026). As shown in Figure 3A, the frequency of non-MI ACS under intensive BP control [1.7% (17 of 1,016)] was significantly greater than that under standard BP control [0.5% (5 of 1,032)] in subgroup II, with an HR of 3.62 (95% CI: 1.33, 9.81; *P* = 0.012). The number needed to harm (NNH) for this subgroup was 84.24 to cause 1 non-MI ACS event (ARR = −1.19%; 95% CI:−2.08,−0.29%). Moreover, for this subgroup, the cumulative hazard of non-MI ACS in the standard arm was significantly lower than that in the intensive arm (Figure 3B), although no significant difference was observed between intensive and standard SBP control in other subgroups.

### External Validation in ACCORD-BP Population

The external validation dataset included 4,495 participants with ACCORD with complete pretreatment factors and covariates, with 2,253 participants in the intensive SBP control group and 2,242 in the standard group [95% CI (4,495/4,733) of the randomized participant sample; Table 1].

When directly applying the trained SOM in the ACCORD, we obtained four subgroups with similar characteristics as the SPRINT (Supplementary Figure 3). Detailed description information for each subgroup was provided in Supplementary Table 2. Supplementary Figure 4 showed an acceptable covariate balance within each subgroup for most of the absolute mean standardized differences were <0.1 and all <0.15. Supplementary Figure 2B showed that consistent with reported results from the ACCORD, participants tend to benefit from intensive BP treatment in most cases, although most of them failed to reach a statistical significance.

We investigated the incidence of primary endpoints within each subgroup from ACCORD too (Supplementary Table 4). Similar to subgroups identified in SPRINT, CVD prevalence was the highest for subgroup I, and subgroup III had a relatively low incidence for most cardiovascular events. It was noteworthy that evidence of HTEs in the ACCORD validated our findings in the SPRINT. As depicted in Supplementary Figure 5A, subgroup II was more likely to experience non-MI ACS in the intensive group (5.4% [31 of 576]) compared with that in the standard group [3.1% (18 of 587)]. In detail, subgroup II had a NNH of 43.19 to cause 1 non-MI ACS event (ARR = −2.32%; 95% CI: −4.63, 0.00%), which meant intensive SBP control did harm to subgroup II [HR = 1.81; 95% CI: 1.01, 3.25; *P* = 0.046]. A statistically significant difference was also revealed by cumulative hazard curves, further indicating the heterogeneity (Supplementary Figure 5B). This independent finding supported our results and interpretation for the multivariate data-driven population subgrouping in assessing the CVD risk for the SPRINT.

### Sensitivity Analysis

The sensitivity analyses showed that the heterogeneity results from the SPRINT and ACCORD remained stable (Supplementary Table 5). Moreover, visualizations based on PCA were shown in Supplementary Figures 6, 7 for SPRINT data, further illustrating that the SOM-based subgroups presented different clinical characteristics. The first three principal components explained about 57.6% of information (variance), and the contribution of each variable to them was consistent with the characteristics of the SOM-based subgroups aforementioned, which confirmed the rationality of the SOM subgroup classification.

## Discussion

This secondary analysis of the SPRINT and ACCORD identified four distinct subgroups with contrasted profiles and prognosis using the SOM method based on individual components of the FRS. It provided evidence on the heterogeneity of intensive BP treatment effects, despite the overall beneficial average treatment effect in the SPRINT trial or no difference of treatment effect in the ACCORD (except for stroke). Our findings suggested that for individuals with T2DM or those nondiabetic patients, most CVD were specifically located within subgroup I; patients with a low prevalence of CVD were distributed across two clusters with varying clinical presentations, especially subgroup III; remarkably, subgroup might be harmed by an intensive intervention for non-MI ACS. These results were of critical importance because the heterogeneity of BP treatment effects on CVD could provide guidance for choosing of BP target and preventing CVD in advance, especially for high-risk patients within subgroup I and HTE for non-MI ACS within subgroup II. To be specific, (i) individuals at a high risk of developing CVD with clinical features of male with low TC and HDL-C levels (subgroup I), should receive more attention in individual primary care; (ii) in particular, patients with high SBP and no antihypertensive agent use at baseline (subgroup II) were more likely to benefit from standard antihypertensive therapy to avoid developing a statistically significant non-MI ACS.

Most patients with CVD were in subgroup I, characterized by male with low TC and HDL-C levels, while fewer patients with CVD were spread across subgroup III that summarized by a profile of female with the highest levels of TC and HDL-C. By a previous study, the prevalence of CVD in men was found higher than that in women, and HDL and TC were also strong, an inverse predictors of CVD risk. Subgroup IV, comprised mostly younger patients with antihypertensive medication use and smoking history, represented the lowest risk for stroke in both the SPRINT and ACCORD, and HF, relatively low risk for death from CVD causes or primary CVD outcome in the SPRINT, which was in-line with previous results from the ACCORD study. Since smoking, age, and high SBP were all independent CVD risk factors, smoking status here seemed to have a more complex role than classical one-variable-at-a-time analysis (24, 25). Finally, patients with the highest SBP and no antihypertensive agents used at baseline visit could benefit from standard treatment to prevent a statistically significant non-MI ACS. As in previous studies, it may be explained by rapid blood pressure reduction. Because of atherosclerosis and arterial aging in middle-aged and older-aged patients with multiple risk factors, the poor vasomotor function could result in insufficient blood pressure autoregulation (26). Appropriate control of blood pressure might not provoke acute myocardial ischemia in these cases. However, among individuals with higher SBP and no antihypertensive agents use, the organs with atherosclerotic stenosis have adapted to a higher level of blood perfusion. An excessive or rapid decrease in blood pressure might cause organic hypoperfusion, aortic blood supply reduction, plaque rupture, blood clot formation, and arterial occlusion that could precipitate myocardial ischemia (27). To sum up, the detailed potential mechanism was hard explored through this secondary analysis, and further researches are warranted.

The SPRINT and ACCORD are the most extensive randomized controlled trials evaluating the clinical effectiveness of intensive SBP control (18). In this context, the strengths of the research included a large sample size; a diverse hypertensive population with a relatively high risk for CVD; their rigor in implementing the protocol; finally, and most importantly, the results being supported in type 2 diabetic individuals or nondiabetic individuals (28). In addition, the extensive theoretical and empirical studies suggested that conventional univariate subgroup analyses were very limited in their ability to detect clinically essential heterogeneities in treatment effects (18). On the contrary, the unsupervised, data driven, SOM-based multivariable analyses in this report might identify individual components of the FRS from the bottom up without the reduction of statistical power such as top–down predefined subanalyses, and avoid false-positive results because of the overfitting of the model by not including the clinical outcomes in the model training phase (13, 29–31). Besides, the SOM had many advantages as follows: (1) expert-driven decision-making based on visualization made up the deficiency of traditional subgrouping method such as K-means for datasets without a clear-clustered structure; (2) there was no limitation to linear relation; (3) statistics incorporated into visualization made it easier to estimate the statistical significance of the morbidity pattern, etc.; and (4) an unsupervised-based subgrouping method was likely to suffer from a large number of noise variables that could be ignored in this work since SOM analysis was based on individual components of the FRS that are all major risk factors for developing CVD (20, 32).

Using the SOM-based method, our analysis of the SPRINT and ACCORD provided evidence for the clinically guiding significance of individual FRS components on cardiovascular outcomes. Our findings suggested that CVD incidence or the benefit of intensive blood pressure intervention varied across individuals, improving the refinement of the clinical practice guidelines on interventional strategies to prevent CVD in the real world. Indeed, further validation or mechanism exploration in prospective studies among more general populations was needed.

## Limitation

Despite these potentially relevant findings, there were also several limitations in the present analysis that require careful consideration. First, we could not dissect the potential mechanism of CVD-risk-specific factors or HTEs captured by SOM analysis since the nature of this study was a secondary *post-hoc* analysis. Second, although participants with the SPRINT and ACCORD were diverse in baseline characteristics, some patients aged younger than 40 years were excluded, etc., and meanwhile, the FRS did not consider some other potential CVD risk factors such as family history of CVD, which may limit the universality of our findings to critical patients (33, 34). Third, the selection of SOM-based subgroup boundaries was subjective, and the border might not be entirely suitable for a particular variable (18).

## Data Availability Statement

Publicly available datasets were analyzed in this study. This data can be found here: 1. SPRINT: https://biolincc.nhlbi.nih.gov/studies/sprint/; 2. ACCORD: https://biolincc.nhlbi.nih.gov/studies/accord.

## Ethics Statement

The studies involving human participants were reviewed and approved by the Internal Review Board of Nanjing Medical University (China). The patients/participants provided their written informed consent to participate in this study.

## Author Contributions

YZ, DY, and YW contributed to the study design. YZ provided the study materials or patients. YW and JB led the data analysis with support from YZ and DY. YW, JB, MZ, FS, and HY drafted the manuscript. YZ and DY performed a critical review of the manuscript. All authors contributed to the critical revision of the manuscript and approved its final version.

## Funding

This study has the following funding sources: The National Natural Science Foundation of China (82173620 to YZ) and the Priority Academic Program Development of Jiangsu Higher Education Institution (PAPD).

## Conflict of Interest

The authors declare that the research was conducted in the absence of any commercial or financial relationships that could be construed as a potential conflict of interest.

## Publisher's Note

All claims expressed in this article are solely those of the authors and do not necessarily represent those of their affiliated organizations, or those of the publisher, the editors and the reviewers. Any product that may be evaluated in this article, or claim that may be made by its manufacturer, is not guaranteed or endorsed by the publisher.
