# Supplementary material for: Heterogeneity of Treatment Effects for Intensive Blood Pressure Therapy by Individual Components of FRS: An Unsupervised Data-Driven Subgroup Analysis in SPRINT and ACCORD
Source: Front Cardiovasc Med. 2022 Feb 3;9:778756. doi: 10.3389/fcvm.2022.778756 (PMC8850629; doi:10.3389/fcvm.2022.778756)
Supplement: Supplementary file 1 [file Data_Sheet_1.docx]

Supplementary Material

# Supplementary Data

SPRINT data included was released on June 24, 2019, available on the site: <https://biolincc.nhlbi.nih.gov/studies/sprint/>. And ACCORD BP data can be applied at https://biolincc.nhlbi.nih.gov/studies/accord/.

# Supplementary Figures and Tables

## Supplementary Figures


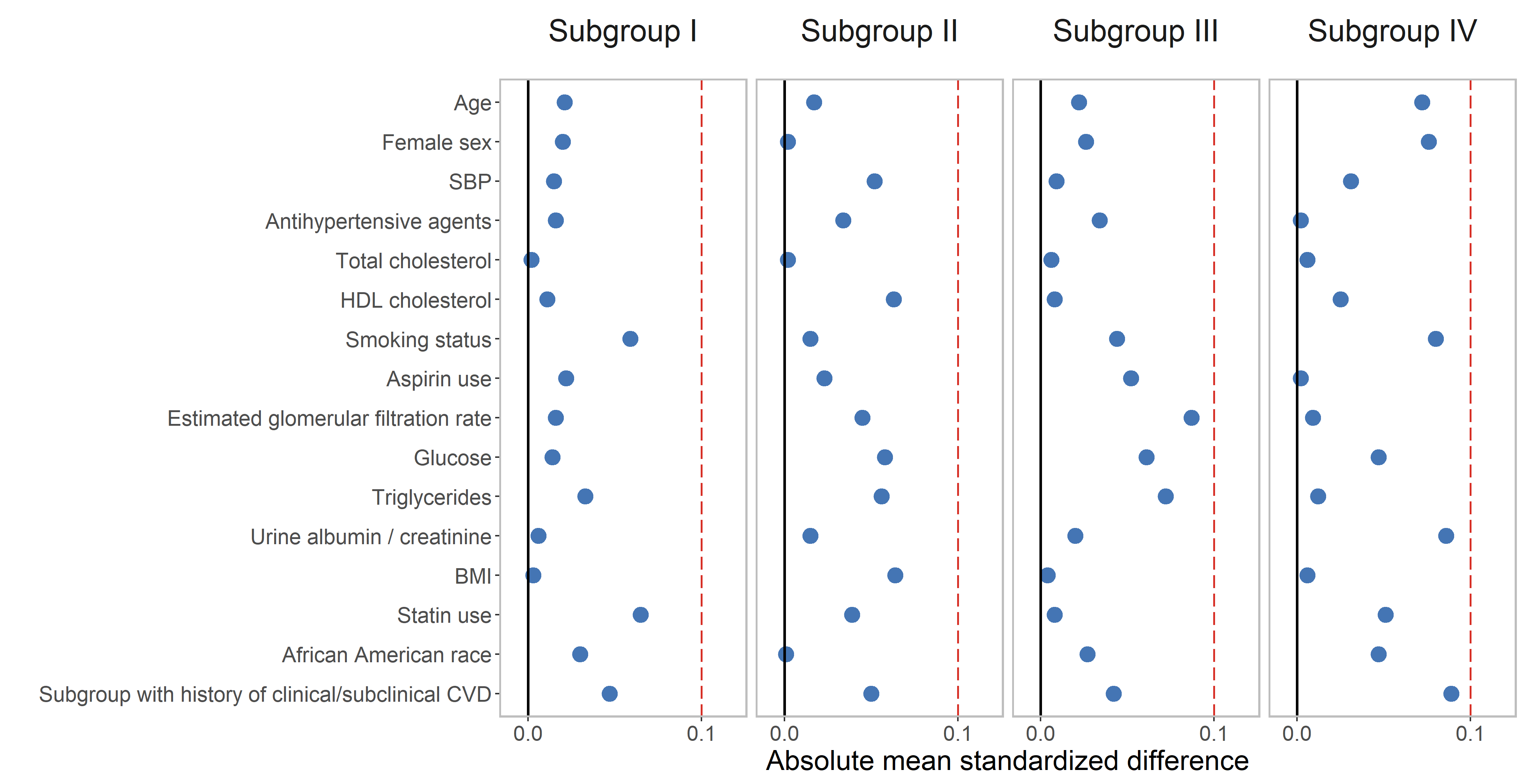


**Supplementary Figure 1.** Scatter Plot Illustrating the Balance in Covariates Between Intensive and Standard Treatment Group in the SPRINT (N = 8,773). Standardized mean difference of 0.1 shown as dashed line. An absolute mean standardized difference indicated good covariate balance. Abbreviations: SPRINT, the systolic blood pressure intervention trial; SBP, systolic blood pressure; HDL, high-density lipoprotein.


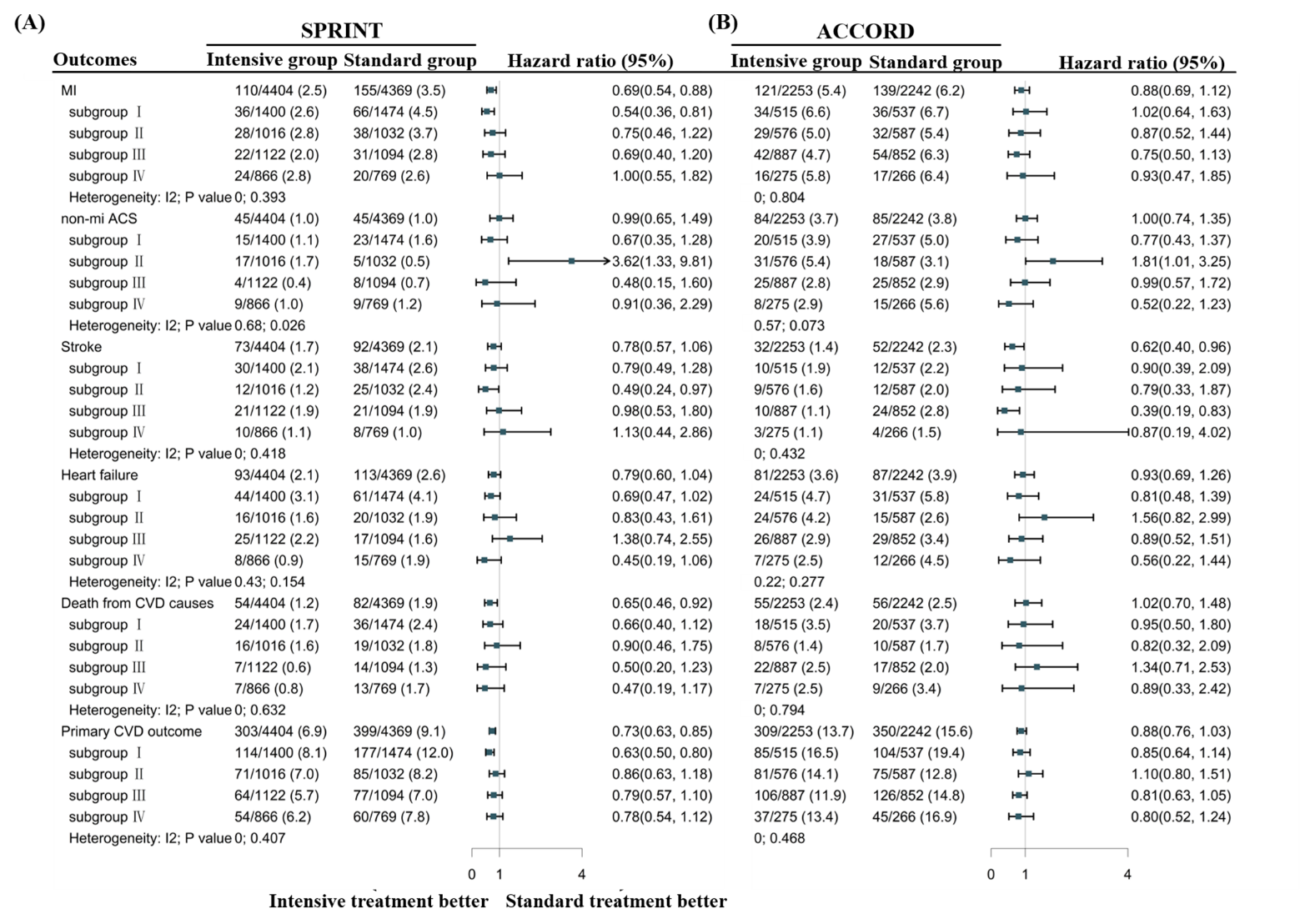


**Supplementary Figure 2.** Forest plots for the primary cardiovascular outcomes in the SPRINT (N = 8,773) and ACCORD (n = 4,495) separately by four SOM-based subgroups. Results from Cox analysis and heterogeneity analysis of overall and four SOM-based subgroups, with adjustment for age, female sex, smoking status, BMI, statin use, and race. HR < 1 denoted that people could benefit from intensive BP treatment, and HR > 1 indicated harm from intensive treatment. 95% CI not crossing 1 indicated a significant association. I^2^ and *P-*value were calculated from Cochran's Q test. Abbreviations: SPRINT, the systolic blood pressure intervention trial; ACCORD, the Action to Control Cardiovascular Risk in Diabetes; HR, hazard ratio; CI, confidence intervals.


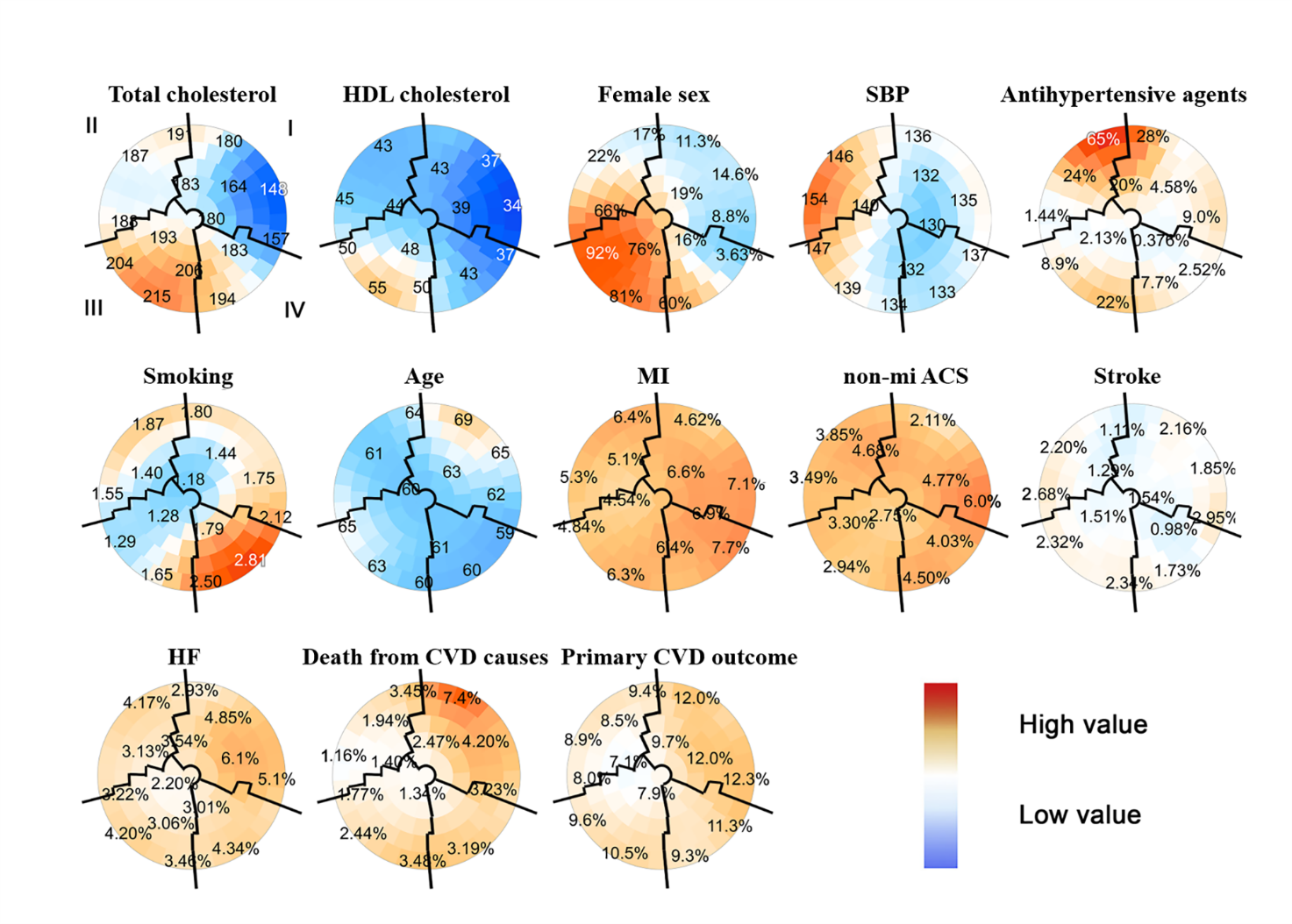


**Supplementary Figure 3.** Subgrouping results from individual components of the FRS using the self-organizing map in the ACCORD (n = 4,495).

Each component plane above showed colouring on the same SOM. The upper part of the two-dimensional SOMs were seven components of the FRS, including total cholesterol, HDL cholesterol, female sex, SBP, antihypertensive agents use, smoking status, and age. Six cardiovascular outcomes included MI, non-mi ACS, stroke, HF, death from CVD causes, and a composite cardiovascular outcome were in the lower part of the figure. The position for each participant on the map was unique and only dependent on the seven individual components of the FRS, so each participant was always located in the same place on each map. The color scale indicates the deviation from population mean with respect to the random fluctuations that could be expected by chance; dark red indicated the highest and dark blue for the lowest values. The numbers on the map represented the local mean value or prevalence (binary variables) for that region. Close districts were selected based on visual identification to provide relevant subgroups of individuals whose boundaries were marked by black lines. A total of four population subgroups were determined: Subgroup I was characterized by men with the lowest TC, while subgroup III was women with the highest mean TC and HDL-C; subgroup II had the highest mean SBP with no antihypertensive medication use at the baseline visit, and subgroup IV characterized by mostly younger patients with antihypertensive medication use and smoking history. Patients with similar components of FRS were located close to each other throughout the map. Abbreviations: ACCORD, the Action to Control Cardiovascular Risk in Diabetes; FRS, Framingham risk score; SOM, self-organizing maps; HDL, high-density lipoprotein cholesterol; SBP, systolic blood pressure; MI, myocardial infarction; non-mi ACS, non-myocardial infarction acute coronary syndrome; HF, heart failure; CVD, cardiovascular disease.


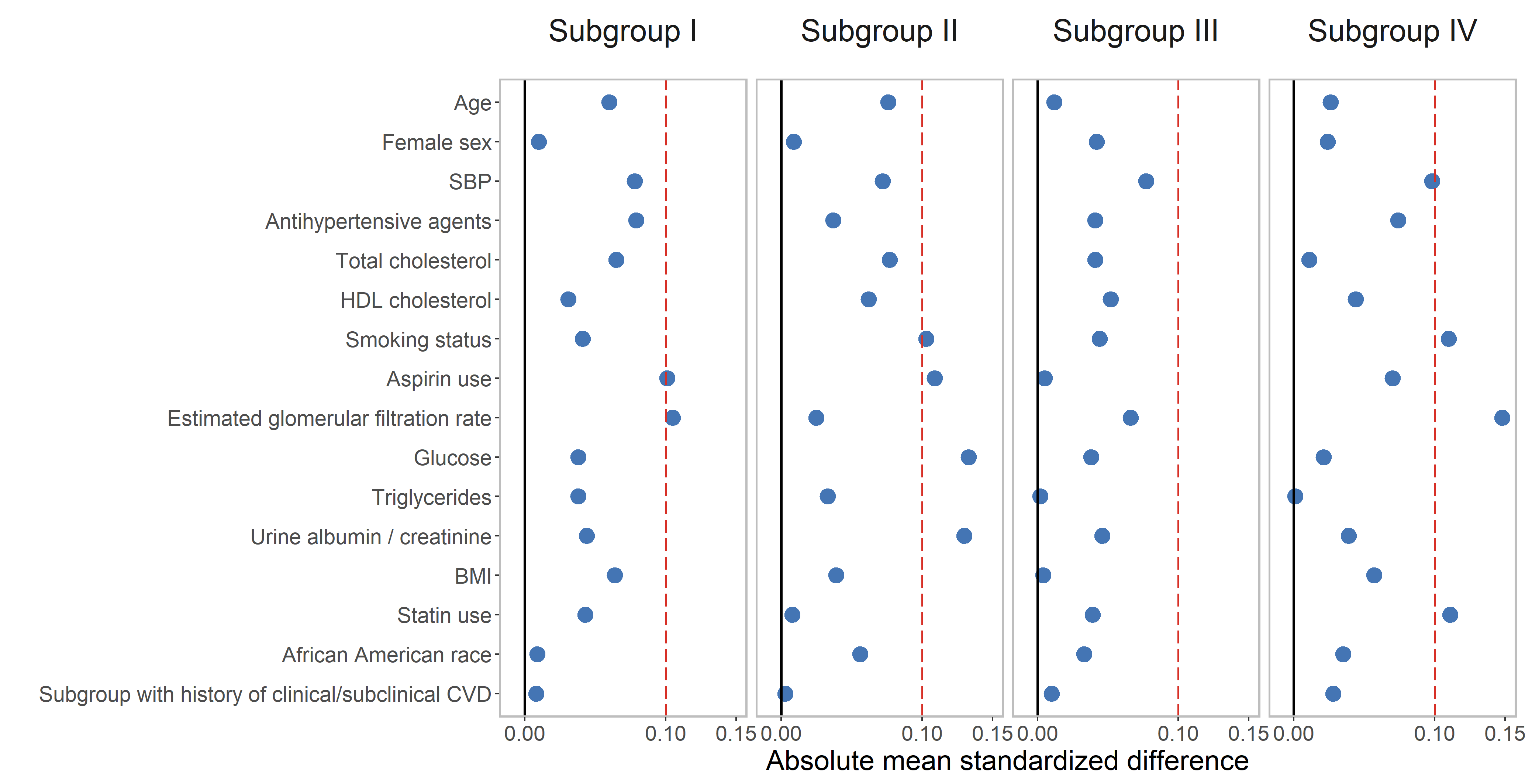


**Supplementary Figure 4.** Scatter Plot Illustrating the Balance in Covariates Between Intensive and Standard Treatment Group in the ACCORD (n = 4,495). Standardized mean difference of 0.1 shown as dashed line. An absolute mean standardized difference indicated good covariate balance. Abbreviations: ACCORD, the Action to Control Cardiovascular Risk in Diabetes; SBP, systolic blood pressure; HDL, high-density lipoprotein.


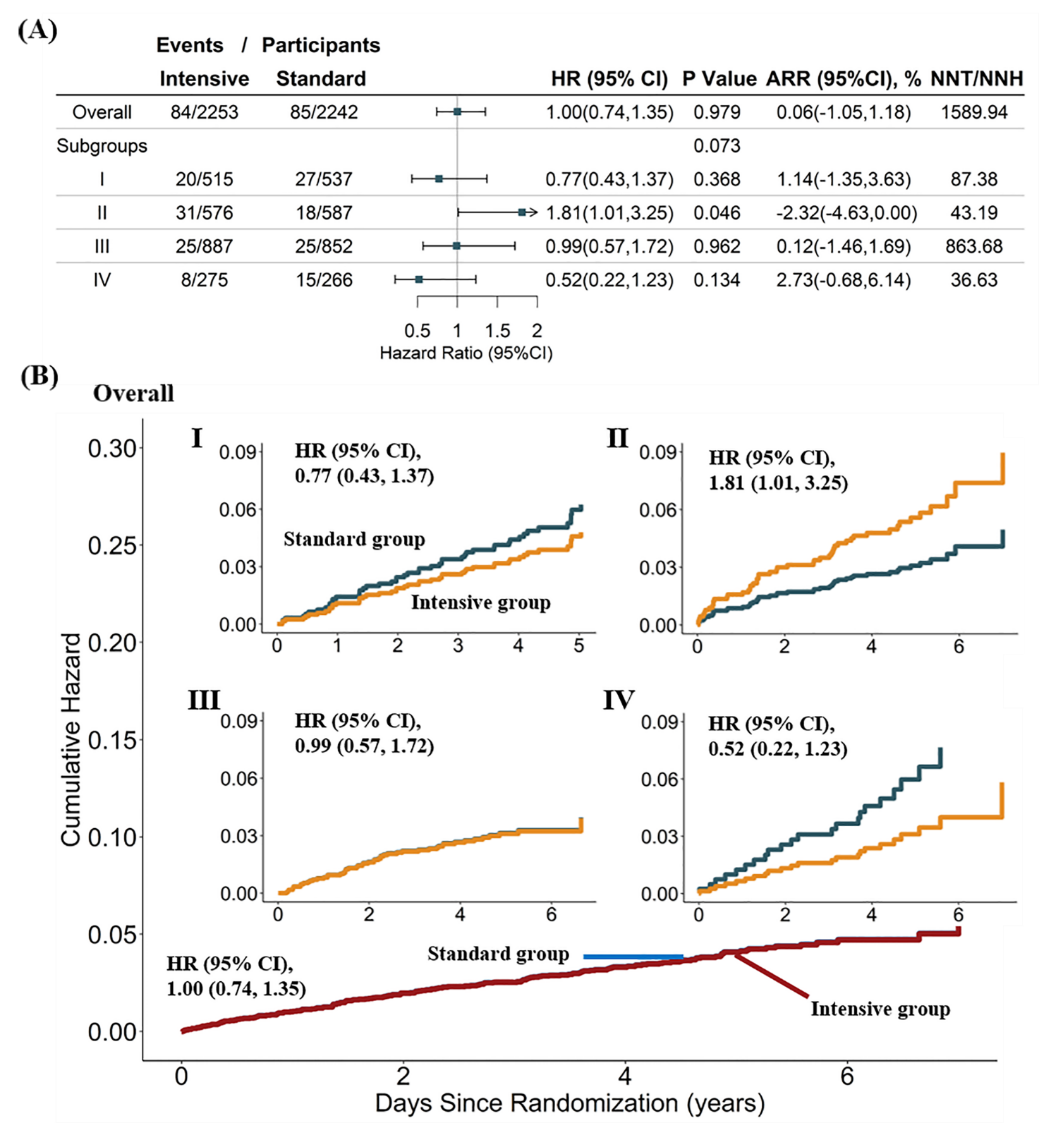


**Supplementary Figure 5.** Heterogeneity analysis and cumulative hazard curves for non-mi ACS in the overall population and four SOM-based subgroups in the ACCORD dataset (n = 4,495). **(A)**, Cox analysis and heterogeneity analysis of four SOM-based subgroups, with adjustment for age, female sex, smoking status, BMI, statin use, and race; absolute risk reduction (ARR) = CVD incidence in the standard-treatment group - CVD incidence in the intensive-treatment group. **(B)**, Cumulative hazard curves across intensive and standard treatment groups. Abbreviations: ACCORD, the Action to Control Cardiovascular Risk in Diabetes; SOM, self-organizing maps; non-mi ACS, non-myocardial infarction acute coronary syndrome; HR, hazard ratio; CI, confidence intervals.


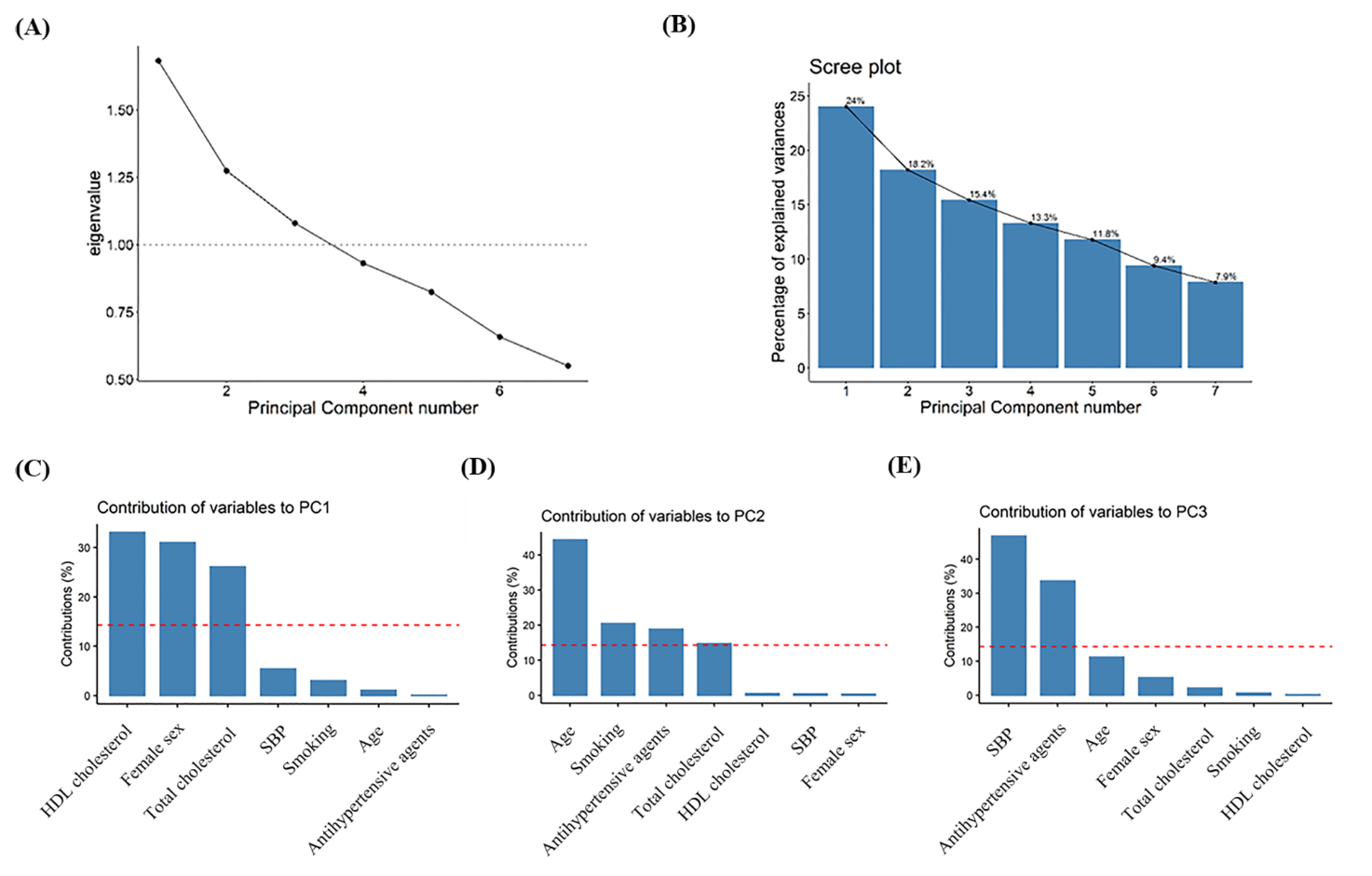


**Supplementary Figure 6.** Visualizations of principal component analysis for SPRINT Data (n = 8,773). **(A)**, The plot of eigenvalue corresponding to 7 individual components of the FRS. An eigenvalue > 1 indicated that principal components (PCs) accounted for more variance than one of the original variables in standardized data. **(B)**, Scree Plot. Eigenvalues were ordered from largest to smallest. From plots **(A)** and **(B)**, we might want to stop at the 3rd principal component since only the first three eigenvalues > 1 and 57.6% of the information (variances) contained in the data were retained by the first three PCs. **(C)**-**(E)**, Bar plots of contributions of the seven components of the FRS to PCs. The red dashed line indicated the expected average contribution, and a variable with a contribution larger than this cutoff could be considered necessary in contributing to the corresponding component. **(C)**-**(E)** showed that female sex, total cholesterol, and HDL cholesterol contributed the most to the 1st PC; age smoking status, and antihypertensive medication use were most essential variables for the 2nd PC; SBP and antihypertensive agent use contributed most to the 3rd PC, which was consistent with the characteristics of the four SOM-based subgroups identified in the SPRINT.


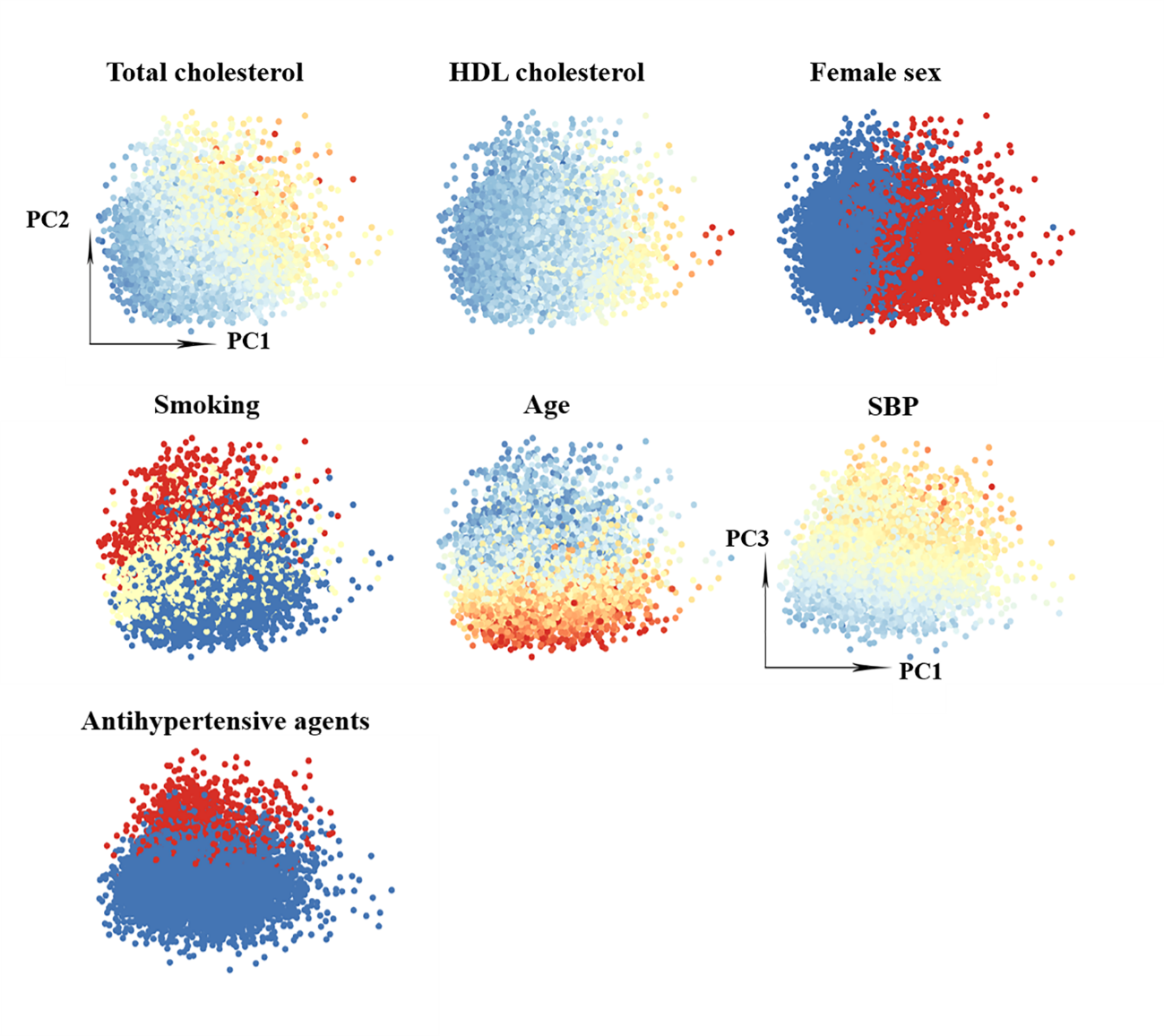


**Supplementary Figure 7.** Scatter plots for the first three principal component scores of SPRINT (n = 8,773). Colors were assigned according to the values of individual components of the FRS: blue indicated a low standardized value (0 = deep blue), and red indicated a high standardized value (1 = deep red). The top five plots, including total cholesterol, HDL cholesterol, female sex, smoking status, and age, were based on the first two principal components (PCs), while the last two (SBP and antihypertensive agents) were based on the 1st and 3rd PCs. A similar pattern of the SOM in the SPRINT was also observed.

## Supplementary Tables

**Supplementary Table 1.** Baseline Characteristics by Four SPRINT Subgroups (n = 8,773).

| **CVD** | **Overall (n=8773)** | **Subgroup I (n=2874)** | **Subgroup II (n=2048)** | **Subgroup III (n=2216)** | **Subgroup IV (n=1635)** | ***P* value** |
| --- | --- | --- | --- | --- | --- | --- |
| Age, mean ± SD, y | 67.90 ± 9.44 | 71.12 ± 9.83 | 66.60 ± 8.67 | 69.04 ± 9.04 | 62.33 ± 7.11 | <0.001* |
| Female sex, n (%) | 3101 (35.3) | 372 (12.9) | 626 (30.6) | 1742 (78.6) | 361 (22.1) | <0.001* |
| SBP, mean ± SD, mm Hg | 139.72 ± 15.63 | 135.02 ± 13.34 | 152.72 ± 14.90 | 138.90 ± 13.97 | 132.80 ± 12.48 | <0.001* |
| Antihypertensive agents, N(%) | 7958 (90.7) | 2705 (94.1) | 1529 (74.7) | 2119 (95.6) | 1605 (98.2) | <0.001* |
| Total cholesterol, mean ± SD, mg/dl | 189.97 ± 41.14 | 164.23 ± 33.09 | 190.61 ± 34.09 | 220.22 ± 39.66 | 193.42 ± 33.97 | <0.001* |
| HDL cholesterol, mean ± SD, mg/dl | 52.79 ± 14.52 | 45.14 ± 10.23 | 51.78 ± 11.13 | 64.44 ± 16.23 | 51.71 ± 12.08 | <0.001* |
| Smoking status, n (%) |  |  |  |  |  |  |
| Never | 3850 (43.9) | 1442 (50.2) | 928 (45.3) | 1326 (59.8) | 154 ( 9.4) | <0.001* |
| Former | 3740 (42.6) | 1288 (44.8) | 935 (45.7) | 796 (35.9) | 721 (44.1) |  |
| Current | 1183 (13.5) | 144 ( 5.0) | 185 ( 9.0) | 94 ( 4.2) | 760 (46.5) |  |
| Aspirin use, n (%) | 4475 (51.0) | 1546 (53.8) | 1143 (55.8) | 1111 (50.1) | 675 (41.3) | <0.001* |
| Estimated glomerular filtration rate, mean ± SD, mL/min/1.73 m^2^ | 71.67 ± 20.67 | 70.09 ± 20.61 | 69.27 ± 20.11 | 74.15 ± 20.27 | 74.09 ± 21.39 | <0.001* |
| Glucose, mean ± SD, mg/dL | 98.89 ± 13.61 | 99.04 ± 13.36 | 99.13 ± 13.78 | 99.42 ± 14.03 | 97.63 ± 13.17 | <0.001* |
| Triglycerides, mean ± SD, mg/dL | 126.05 ± 84.04 | 127.68 ± 85.95 | 124.25 ± 74.65 | 126.55 ± 76.74 | 124.79 ± 99.74 | 0.482 |
| Urine albumin in mg/(creatinine in g × 0.01) , mean ± SD | 42.48 ± 166.51 | 53.12 ± 204.75 | 48.87 ± 177.04 | 27.87 ± 98.42 | 35.58 ± 148.81 | <0.001* |
| Body mass index, mean ± SD, kg/m^2^ | 29.88 ± 5.79 | 30.00 ± 5.37 | 30.28 ± 5.95 | 29.62 ± 6.18 | 29.55 ± 5.69 | <0.001* |
| Statin use, n (%) | 4920 (56.1) | 1434 (49.9) | 1018 (49.7) | 1300 (58.7) | 1168 (71.4) | <0.001* |
| African American race, n (%) | 2800 (31.9) | 1015 (35.3) | 655 (32.0) | 588 (26.5) | 542 (33.1) | <0.001* |
| Subgroup with a history of clinical/subclinical CVD, n (%) | 1788 (20.4) | 673 (23.4) | 470 (22.9) | 449 (20.3) | 196 (12.0) | <0.001* |

Note. Values were described as mean ± SD or number (%). *P* values were based on ANOVA or Pearson's chi-squared tests. **P*-value < 0.05.

Abbreviations: SPRINT, the systolic blood pressure intervention trial; SBP, systolic blood pressure; HDL, high-density lipoprotein; SD, standard deviation; SMD, standardized mean difference.

**Supplementary Table 2.** Baseline Characteristics by Four ACCORD Subgroups (n = 4,495).

| **CVD** | **Overall (n=4495)** | **Subgroup I (n=1052)** | **Subgroup II (n=1163)** | **Subgroup III (n=1739)** | **Subgroup IV (n=541)** | ***P* value** |
| --- | --- | --- | --- | --- | --- | --- |
| Age, mean ± SD, y | 62.77 ± 6.70 | 64.67 ± 7.08 | 62.27 ± 6.39 | 62.83 ± 6.50 | 59.94 ± 6.06 | <0.001* |
| Female sex, n (%) | 2128 (47.3) | 129 (12.3) | 380 (32.7) | 1435 (82.5) | 184 (34.0) | <0.001* |
| SBP, mean ± SD, mm Hg | 139.24 ± 15.79 | 132.82 ± 13.68 | 149.77 ± 14.92 | 138.54 ± 14.64 | 131.33 ± 12.61 | <0.001* |
| Antihypertensive agents, N(%) | 3868 (86.1) | 956 (90.9) | 826 (71.0) | 1574 (90.5) | 512 (94.6) | <0.001* |
| Total cholesterol, mean ± SD, mg/dl | 192.68 ± 44.53 | 160.02 ± 32.22 | 190.72 ± 39.93 | 212.66 ± 42.44 | 196.21 ± 43.22 | <0.001* |
| HDL cholesterol, mean ± SD, mg/dl | 46.13 ± 13.57 | 37.35 ± 9.82 | 44.41 ± 10.88 | 53.24 ± 13.98 | 44.03 ± 11.74 | <0.001* |
| Smoking status, n (%) |  |  |  |  |  |  |
| Never | 2010 (44.7) | 472 (44.9) | 477 (41.0) | 1050 (60.4) | 11 ( 2.0) | <0.001* |
| Former | 1889 (42.0) | 502 (47.7) | 579 (49.8) | 617 (35.5) | 191 (35.3) |  |
| Current | 596 (13.3) | 78 ( 7.4) | 107 ( 9.2) | 72 ( 4.1) | 339 (62.7) |  |
| Aspirin use, n (%) | 2351 (52.3) | 553 (52.6) | 671 (57.7) | 853 (49.1) | 274 (50.6) | <0.001* |
| Estimated glomerular filtration rate, mean ± SD, mL/min/1.73 m^2^ | 91.50 ± 28.94 | 90.61 ± 23.29 | 86.32 ± 24.51 | 95.13 ± 34.53 | 92.69 ± 26.13 | <0.001* |
| Glucose, mean ± SD, mg/dL | 174.79 ± 57.84 | 168.64 ± 56.75 | 176.92 ± 62.27 | 177.52 ± 54.20 | 173.42 ± 60.50 | 0.001* |
| Triglycerides, mean ± SD, mg/dL | 193.82 ± 176.44 | 188.49 ± 139.79 | 195.69 ± 196.56 | 190.57 ± 145.84 | 210.56 ± 262.81 | 0.088 |
| Urine albumin in mg/(creatinine in g × 0.01) , mean ± SD | 93.39 ± 326.73 | 82.43 ± 253.71 | 138.69 ± 427.26 | 65.03 ± 276.14 | 108.50 ± 342.68 | <0.001* |
| Body mass index, mean ± SD, kg/m^2^ | 32.17 ± 5.47 | 31.45 ± 4.89 | 32.11 ± 5.33 | 32.77 ± 5.77 | 31.78 ± 5.63 | <0.001* |
| Statin use, n (%) | 2934 (65.3) | 756 (71.9) | 850 (73.1) | 1030 (59.2) | 298 (55.1) | <0.001* |
| African American race, n (%) | 1071 (23.8) | 298 (28.3) | 293 (25.2) | 384 (22.1) | 96 (17.7) | <0.001* |
| Subgroup with a history of clinical/subclinical CVD, n (%) | 1523 (33.9) | 380 (36.1) | 405 (34.8) | 537 (30.9) | 201 (37.2) | 0.006* |

Note. Values were described as mean ± SD or number (%). *P* values were based on ANOVA or Pearson's chi-squared tests. **P*-value < 0.05.

Abbreviations: ACCORD, the Action to Control Cardiovascular Risk in Diabetes; SBP, systolic blood pressure; HDL, high-density lipoprotein; SD, standard deviation, standardized mean difference.

**Supplementary Table 3.** Incidence of Primary CVD in the SPRINT by Subgroups (n = 8,773).

| **CVD** | **Overall (n=8773)** | **Subgroup I (n=2874)** | **Subgroup II (n=2048)** | **Subgroup III (n=2216)** | **Subgroup IV (n=1635)** | ***P* value** |
| --- | --- | --- | --- | --- | --- | --- |
| MI | 265 (3.0) | 102 (3.5) | 66 (3.2) | 53 (2.4) | 44 (2.7) | 0.085 |
| Non-mi ACS | 90 (1.0) | 38 (1.3) | 22 (1.1) | 12 (0.5) | 18 (1.1) | 0.052 |
| Stroke | 165 (1.9) | 68 (2.4) | 37 (1.8) | 42 (1.9) | 18 (1.1) | 0.028* |
| Heart failure | 206 (2.3) | 105 (3.7) | 36 (1.8) | 42 (1.9) | 23 (1.4) | <0.001* |
| Death from CVD causes | 136 (1.6) | 60 (2.1) | 35 (1.7) | 21 (0.9) | 20 (1.2) | 0.007* |
| Primary CVD outcome | 702 (8.0) | 291 (10.1) | 156 (7.6) | 141 (6.4) | 114 (7.0) | <0.001* |

Note. Values were number (%). *P* values were based on Pearson's chi-squared tests. **P*-value < 0.05.

Abbreviations: SPRINT, the systolic blood pressure intervention trial; CVD, cardiovascular disease; MI, myocardial infarction; non-mi ACS, non-myocardial infarction acute coronary syndrome.

**Supplementary Table 4.** Incidence of Primary CVD in the ACCORD by Subgroups (n = 4,495).

| **CVD** | **Overall (n=4495)** | **Subgroup I (n=1052)** | **Subgroup II (n=1163)** | **Subgroup III (n=1739)** | **Subgroup IV (n=541)** | ***P* value** |
| --- | --- | --- | --- | --- | --- | --- |
| MI | 260 (5.8) | 70 (6.7) | 61 (5.2) | 96 (5.5) | 33 (6.1) | 0.493 |
| Non-mi ACS | 169 (3.8) | 47 (4.5) | 49 (4.2) | 50 (2.9) | 23 (4.3) | 0.101 |
| Stroke | 84 (1.9) | 22 (2.1) | 21 (1.8) | 34 (2.0) | 7 (1.3) | 0.716 |
| Heart failure | 168 (3.7) | 55 (5.2) | 39 (3.4) | 55 (3.2) | 19 (3.5) | 0.034* |
| Death from CVD causes | 111 (2.5) | 38 (3.6) | 18 (1.5) | 39 (2.2) | 16 (3.0) | 0.013* |
| Primary CVD outcome | 659 (14.7) | 189 (18.0) | 156 (13.4) | 232 (13.3) | 82 (15.2) | 0.004* |

Note. Values were number (%). *P* values were based on Pearson's chi-squared tests. **P*-value < 0.05.

Abbreviations: ACCORD, the Action to Control Cardiovascular Risk in Diabetes; CVD, cardiovascular disease; MI, myocardial infarction; non-mi ACS, non-myocardial infarction acute coronary syndrome.

**Supplementary Table 5.** Sensitivity analysis among identified SOM-based SPRINT subgroups (n = 8,773) and ACCORD subgroups (n = 4,495).

|  | **Model** | **Evaluation method** |  | **Events/Participants** | | **HR (95% CI)** | ***P* value** |
| --- | --- | --- | --- | --- | --- | --- | --- |
|  |  |  |  | Intensive | Intensive |  |  |
| SPRINT (n = 8,773) | 1 | Unadjusted for other covariates | Overall | 45/4404 | 45/4369 | 0.99(0.65, 1.49) | 0.949 |
|  |  |  | Subgroups |  |  |  |  |
|  |  |  | I | 15/1400 | 23/1474 | 0.67(0.35, 1.29) | 0.229 |
|  |  |  | II | 17/1016 | 5/1032 | 3.55(1.31, 9.61) | 0.013* |
|  |  |  | III | 4/1122 | 8/1094 | 0.48(0.15, 1.61) | 0.236 |
|  |  |  | IV | 9/866 | 9/769 | 0.88(0.35, 2.23) | 0.792 |
|  | 2 | Additionally adjustment for total cholesterol, HDL-cholesterol and triglycerides | Overall | 45/4404 | 45/4369 | 0.99(0.65, 1.5) | 0.962 |
|  |  |  | Subgroups |  |  |  |  |
|  |  |  | I | 15/1400 | 23/1474 | 0.67(0.35, 1.28) | 0.226 |
|  |  |  | II | 17/1016 | 5/1032 | 3.42(1.26, 9.29) | 0.016* |
|  |  |  | III | 4/1122 | 8/1094 | 0.48(0.14, 1.58) | 0.227 |
|  |  |  | IV | 9/866 | 9/769 | 0.88(0.35, 2.23) | 0.792 |
| ACCORD (n = 4,495) | 1 | Unadjusted for other covariates | Overall | 84/2253 | 85/2242 | 1(0.74, 1.35) | 0.977 |
|  |  |  | Subgroups |  |  |  |  |
|  |  |  | I | 20/515 | 27/537 | 0.78(0.44, 1.38) | 0.389 |
|  |  |  | II | 31/576 | 18/587 | 1.78(1, 3.19) | 0.051 |
|  |  |  | III | 25/887 | 25/852 | 0.97(0.56, 1.69) | 0.913 |
|  |  |  | IV | 8/275 | 15/266 | 0.53(0.22, 1.26) | 0.149 |
|  | 2 | Additionally adjustment for total cholesterol, HDL-cholesterol and triglycerides | Overall | 84/2253 | 85/2242 | 1(0.74, 1.36) | 0.989 |
|  |  |  | Subgroups |  |  |  |  |
|  |  |  | I | 20/515 | 27/537 | 0.79(0.44, 1.41) | 0.425 |
|  |  |  | II | 31/576 | 18/587 | 1.8(1, 3.21) | 0.049* |
|  |  |  | III | 25/887 | 25/852 | 0.97(0.56, 1.69) | 0.919 |
|  |  |  | IV | 8/275 | 15/266 | 0.53(0.22, 1.25) | 0.144 |

Note. HRs and *P* values were based on Cox proportional hazards regression models adjusted for different covariates. **P*-value < 0.05.

Abbreviations: HR, hazard ratio; CI, confidence intervals.
